# Melatonin modulation of crosstalk among malignant epithelial, endothelial and adipose cells in breast cancer (Review)

**DOI:** 10.3892/ol.2014.2203

**Published:** 2014-05-30

**Authors:** SAMUEL COS, VIRGINIA ÁLVAREZ-GARCÍA, ALICIA GONZÁLEZ, CAROLINA ALONSO-GONZÁLEZ, CARLOS MARTÍNEZ-CAMPA

**Affiliations:** Department of Physiology and Pharmacology, School of Medicine, University of Cantabria and Valdecilla Research Institute, Santander 39011, Spain

**Keywords:** melatonin, pineal gland, endothelial cell, fibroblast, aromatase, MCF-7

## Abstract

Melatonin, the main secretory product of the pineal gland, is an oncostatic agent that reduces the growth and development of various types of tumors, particularly mammary tumors whose growth is dependent on estrogens. Previous *in vivo* and *in vitro* studies point to the hypothesis that melatonin interplays with estrogen signaling pathways at three different levels: i) an indirect mechanism, by interfering with the hypothalamic-pituitary-reproductive axis in such way that the level of plasma estrogens synthesized by the gonadal glands are downregulated; ii) a direct mechanism of the pineal gland at the cell cancer level, disrupting the activation of estradiol receptors, therefore behaving as a selective estrogen receptor modulator; and iii) by regulating the enzymes involved in the biosynthesis of estrogens in other tissues, thus behaving as a selective estrogen enzyme modulator. The intratumoral metabolism and synthesis of estrogens, as a result of the interactions of various enzymes, is more important than blood uptake to maintain mammary gland estrogen levels in menopausal females. Additionally, estrogens are considered to play an important role in the pathogenesis and development of hormone-dependent breast carcinoma. Paracrine interactions among malignant epithelial cells and proximal adipose and endothelial cells, through cytokines and growth factors produced by breast tumor cells, modulate estrogen production at the mammary tumor level and, as a consequence, the genesis and development of mammary tumors. The aim of the present review is to summarize the recent findings describing the mechanisms by which melatonin is able to modulate the crosstalk among malignant epithelial, endothelial and adipose cells in breast cancer.

## 1. Introduction

The oncostatic effects of melatonin are particularly relevant to hormone-dependent tumors (1–5). Of these neoplasias, the most deeply studied have been mammary adenocarcinomas. Based on the role of the pineal gland in inhibiting gonadal maturation and sex hormone secretion in mammals, Cohen *et al* (1978) introduced the hypothesis that a decrease in pineal function decreases melatonin levels and induces a relative ‘hyperestrogenism’, which underlies the development of breast cancer (6). Since then, there has been evidence supporting the theory that the antitumor actions of melatonin in hormone-dependent tumors are mainly based on the antiestrogenic properties of melatonin (5,7).

The oncostatic effects of melatonin in hormone-dependent breast cancer were firstly explained by indirect neuroendocrine mechanisms, such as the downregulation of the neuroendocrine reproductive axis by melatonin, and the consequent reduction of estrogenic hormones responsible for the normal and pathological growth of the mammary gland (8). In addition, it has also been demonstrated that melatonin may directly interfere with the activation of the estrogen receptor and counteract the effects of estrogens at the tumor cell level, thus behaving as a selective estrogen receptor modulator (7,9–11). In more recent years, a third neuroendocrine mechanism has been described in which melatonin is able to reduce the estrogen-mediated development of breast cancer, involving the regulation of certain enzymes responsible for the local synthesis of estrogens, thus behaving as a selective estrogen enzyme modulator (12–15).

## 2. Local synthesis of estrogens in breast cancer epithelial cells and melatonin

**Figure 1:**
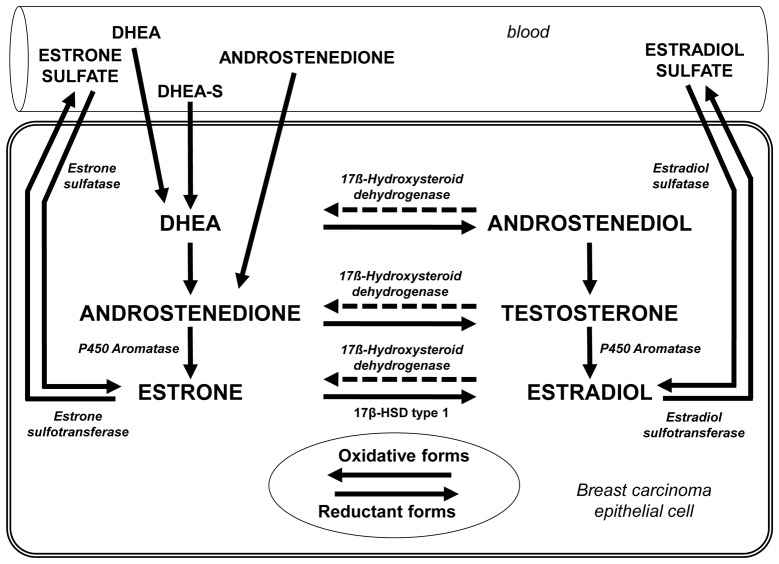
Enzymatic mechanisms involved in the local production of estrogens in human breast carcinoma tissues. DHEA, dehydroepiandrosterone; DHEA-S, DHEA sulfate; *17β-HSD type 1*, 17β-hydroxysteroid dehydrogenase type 1.

The intratumoral metabolism and synthesis of estrogens, as a result of the interactions of various enzymes, is considered to play an important role in the pathogenesis and development of hormone-dependent breast carcinoma (16–19). In breast cancer, particularly that of postmenopausal women, estrogens are synthesized in the mammary tissue by transformation either from androgen precursors, mainly of adrenal origin, or from biologically inactive estrogens. Breast carcinoma epithelial cells contain all the enzymes necessary for the local synthesis of estrogens (Fig. 1). One of the major pathways involved in the synthesis of estrogens in breast cancer cells is the aromatase pathway, which transforms androgens into estrogens (20). Aromatase activity and expression is markedly higher in breast cancer tissue than in normal mammary tissue (21,22). The second pathway involved in estrogen formation is the sulfatase pathway, which converts estrogen sulfates into estrone and estradiol (18,19,22). The final step of steroidogenesis in peripheral tissues is the conversion of the weak estrone to the potent biologically active estradiol by the action of the 17β-hydroxysteroid dehydrogenase activity type 1 (17β-HSD1) (18,19). In breast cancer tissue, estrogen sulfotransferase is also present, which converts estrogens into estrogen sulfates. Since the sulfo-conjugated estrogens are the biologically inactive forms of the estrogens, another possible way to control the tissular concentration of active estradiol is to identify new ways to stimulate the enzymes involved in the sulfate formation (19,22).

**Figure 2:**
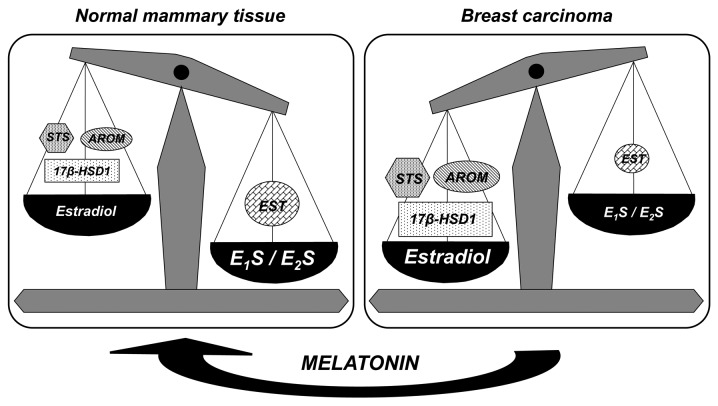
Expression of enzymes associated with the local production of estrogens in human normal mammary tissue and breast carcinoma tissue. In the breast carcinoma tissue, STS, AROM and 17β-HSD1 tend to be overexpressed, while EST is decreased. Melatonin decreases the expression of AROM, STS and 17β-HSD1, and increases the EST expression. Thus, melatonin tends to modify the expression of the enzymes involved in the local synthesis of estrogens, causing it to be similar to the expression of enzymes in the mammary normal tissue. Figure modified from Cos *et al* (23). STS, sulfatase; AROM, aromatase; 17β-HSD1, 17β-hydroxysteroid dehydrogenase type 1; EST, estrogen sulfotransferase; E_1_S, estrone sulfate; E_2_S, estradiol sulfate.

In normal breast tissue, there is a high concentration of circulating inactive steroids, which are the major precursor substrates of local estrogen production, mainly estrone. In this tissue, the estrogen sulfotransferase activity and expression, the enzyme that inactivates estrone and 17β-estradiol, tend to be increased. However, in breast carcinoma tissue, aromatase (which converts androgens into estrogens), sulfatase (which hydrolyzes the estrone sulfates to estrone) and 17β-HSD1 (which converts the estrone to the potent 17β-estradiol) tend to be overexpressed, whereas the expression of estrogen sulfotransferase is frequently decreased, which may result in the accumulation of 17β-estradiol in breast cancer tissues (Fig. 2). At the tumor cell level, melatonin decreases the activity and expression of aromatase, sulfatase and 17β-HSD1, and increases the activity and expression of estrogen sulfotransferase (12,13,23,24). Melatonin tends to modify the activity and expression of the enzymes involved in the local synthesis of estrogens, causing them to be similar to the expression of enzymes in the mammary normal tissue, and may thus protect mammary tissue from excessive estrogenic effects (Fig. 2).

Regulation of aromatase expression in human tissues is relatively complex, involving alternative promoter sites that provide tissue-specific control. In the normal breast, the mammary adipose tissue maintains low levels of aromatase expression almost exclusively via promoter I.4 (25). However, in mammary cancer, both in malignant epithelial cells and fibroblasts, the expression of aromatase is increased via activation of promoters II and I.3, which are regulated by cyclic adenosine monophosphate (cAMP) and factors that regulate cAMP levels (26). Prostaglandin E_2_ (PGE_2_) is an important regulator of aromatase gene expression via promoters II and I.3 (25–28). The formation of PGE_2_ occurs through the activity of the cyclooxygenases (COXs), rate-limiting enzymes that catalyze the conversion of arachidonic acid to prostaglandins. Promoter I.3 and II are considered to be the major promoters driving aromatase expression in breast cancer and surrounding adipose tissue. One of the mechanisms through which melatonin modulates aromatase enzyme in breast tumor cells is through its downregulatory action on the expression of COX enzymes, COX-1 and COX-2, which decrease the levels of PGE_2_. Lower levels of PGE_2_ result in decreased intracellular levels of cAMP, which in turn diminish the activation of promoters I.3 and II, and result in decreased aromatase expression (29,30).

In addition, the antiaromatase and antisulfatase effects of melatonin have also been shown in cancer cell types other than breast cancer cells. In glioblastoma cells, which express estrogen receptors and have the ability to synthesize estrogens, melatonin also reduces the local production of estrogens by decreasing the activity of aromatase, sulfatase and 17β-HSD1, and downregulating aromatase, sulfatase and 17β-HSD1 mRNA steady state levels (31,32).

This melatonin modulatory effect on the aromatase and sulfatase enzymes, at the tumor cell level, has also been described *in vivo*, in rats bearing 7,12-dimethylbenzanthracene-induced mammary tumors. The growth of these mammary tumors is estrogen-dependent and ovariectomy significantly reduces both the size and number of tumors, while the administration of testosterone or estrone sulfate to ovariectomized animals is able to maintain the tumor growth at the same level as the control (uncastrated) animals. The stimulatory effects of tumor development induced by testosterone (which depends on the local synthesis of estrogens from androgens), due to the aromatase action, or estrone sulfate (which depends on the estrogens locally formed by the action of the sulfatase enzyme on the biologically inactive estrogens), are suppressed by the administration of melatonin. Tumors from animals treated with melatonin have the lowest microsomal aromatase and sulfatase activity (14,33).

## 3. Local synthesis of estrogens in peritumoral fibroblasts and melatonin

**Figure 3:**
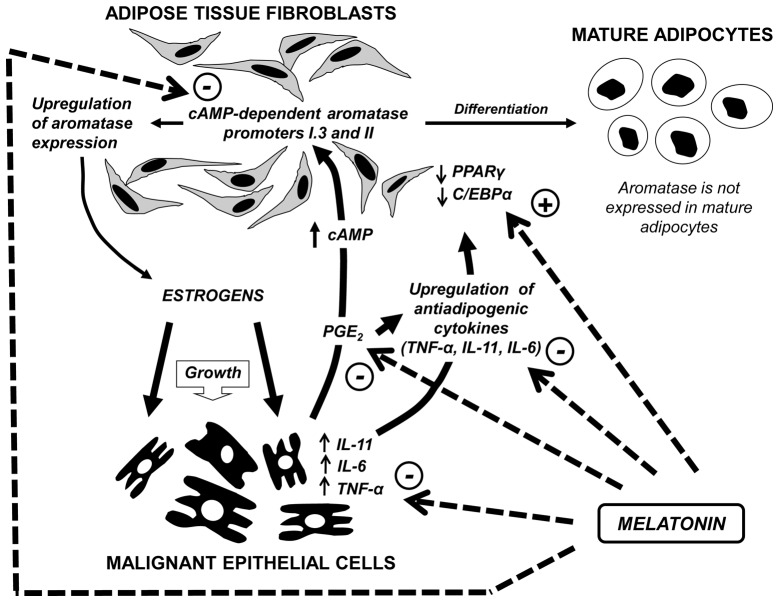
Epithelial-stromal interactions in breast tumors inhibit adipogenic differentiation and enhance estrogen formation by increasing the aromatase activity of the undifferentiated fibroblasts. All these actions are mediated by cytokines, such as TNF-α, IL-6 and IL-11, produced by malignant epithelial cells. Melatonin reduces the formation of undifferentiated fibroblasts surrounding malignant epithelial cells by stimulating the differentiation of fibroblasts to mature adipocytes and adipogenesis, and by decreasing the aromatase activity of the fibroblasts and adipocytes through a downregulatory action on the expression of antiadipogenic cytokines, which decreases the levels of these cytokines. Lower levels of TNF-α, IL-6 and IL-11 stimulate the differentiation of fibroblasts and decrease the aromatase activity and expression. Melatonin also decreases the production of PGE_2_ by malignant cells, which upregulates aromatase expression both in the tumor itself and in the surrounding adipose tissue, and enhances the production of IL-11 by tumor cells. Figure modified from Álvarez-García *et al* (39) and Cos *et al* (23). TNF-α, tumor necrosis factor-α; IL, interleukin.

In breast tumors, the majority of aromatase and sulfatase activity and expression, the two principal pathways of synthesis of estrogens, are found in the fibroblast component of the adipose tissue and in vascular endothelial cells. The local biosynthesis of estrogens in breast cancer depends on paracrine interactions between malignant epithelial cells and proximal fibroblasts and vascular endothelial cells. Malignant epithelial cells secrete cytokines, including tumor necrosis factor α (TNF-α), interleukin 6 (IL-6) and IL-11, which are upregulated by estrogens. These cytokines inhibit the differentiation of surrounding fibroblasts into mature adipocytes, through the selective inhibition of expression of peroxisome proliferator-activated receptor γ (PPARγ) and CCAAT/enhancer binding protein α (C/EBPα), and also stimulate aromatase expression in these undifferentiated fibroblasts (Fig. 3) (19,34,35). This biological phenomenon is commonly known as the desmoplastic reaction or the accumulation of undifferentiated fibroblasts with high aromatase activity surrounding malignant epithelial cells. Tumor cells also secrete other factors, such as PGE_2_, which stimulate aromatase activity and expression in these undifferentiated fibroblasts, as well as upregulating antiadipogenic cytokines.

3T3-L1 is a fibroblast cell line that is initially fibroblastic but which, under appropriate conditions, differentiates into adipocytes (36). Melatonin treatment during the preadipocyte differentiation enhances the adipogenesis, and higher doses of melatonin induce more extensive deposits of lipid droplets and also induce a ~50% reduction in the aromatase activity of the cells, two indicators of adipogenic differentiation. It has been demonstrated that melatonin significantly increases the expression of PPARγ and C/EBPα, the two main regulators of terminal adipogenesis (37). An approach to simulate *in vitro* the situation occurring in the mammary tumor is to use cocultures of malignant epithelial cells with fibroblasts or endothelial cells. The presence of malignant epithelial cells in the cocultures inhibits the differentiation of preadipocytes to adipocytes and reduces the intracytoplasmic triglyceride accumulation, an indicator of adipogenic differentiation. The presence of malignant cells also stimulates the aromatase activity in the fibroblasts. Melatonin counteracts the inhibitory effect on adipocyte differentiation induced by malignant epithelial cells, and also counteracts the stimulatory effect of the presence of breast cancer cells on aromatase activity in fibroblasts (37–39).

The levels of antiadipogenic cytokines, TNF-α, IL-6 and IL-11, in the coculture media are 10-fold higher than those found in the culture of fibroblasts alone, since epithelial malignant cells, in the presence of fibroblasts, secrete these cytokines with the aim to inhibit the differentiation of preadipocytes into adipocytes and to accumulate undifferentiated fibroblasts with high aromatase activity around malignant epithelial cells. The addition of melatonin to the cocultures decreases the concentrations of cytokines in the media and counteracts the stimulatory effect induced by the presence of malignant cells on the cytokines levels. Melatonin also induces a reduction in the TNF-α, IL-6 and IL-11 mRNA expression in breast cancer epithelial cells and fibroblasts (Fig. 3). The addition of luzindole, a melatonin receptor antagonist, prevents this inhibitory effect of melatonin on cytokines expression, indicating that melatonin acts through known melatonin receptor-mediated mechanisms (39).

In summary, melatonin may reduce the level of undifferentiated fibroblasts surrounding malignant epithelial cells by stimulating the differentiation of fibroblasts to mature adipocytes and adipogenesis, and by decreasing the aromatase activity of the fibroblasts through a downregulatory action on the expression of antiadipogenic cytokines, which decreases the levels of these cytokines. Lower levels of TNF-α, IL-6 and IL-11 allow the differentiation of fibroblasts, as well as decreasing the aromatase activity and expression. Melatonin also decreases the production of PGE_2_ by malignant cells, which downregulates aromatase expression and cytokine production in the tumor itself and in the surrounding adipose tissue. Lower levels of aromatase lead to lower levels of estrogens, resulting in decreased growth and development of the breast tumor (Fig. 3).

## 4. Local synthesis of estrogens in peritumoral endothelial cells and melatonin

Endothelial cells also represent a critical cellular element in the tumor microenvironment, which play a crucial role in the growth and progression of breast tumors. They are another source of estrogens, as they also express aromatase (40,41). Promoter I.7 is a novel breast cancer-associated aromatase promoter mainly active in vascular endothelial cells, and is upregulated in breast cancer tissue (42). Excessive aromatase expression via promoters I.3, II and I.7, and consequent increase in estrogen biosynthesis in malignant epithelial cells, undifferentiated adipose fibroblasts and adjacent endothelial cells contribute to the development and progression of breast cancer. In addition, endothelial cells provide structural and biochemical support for tumor growth and progression of cancer through control of angiogenesis. Vascular endothelial growth factor (VEGF) secreted by breast cancer cells is essential for the expansion of breast cancer and may function in both paracrine and autocrine manners to promote the proliferation, growth, survival and migration of endothelial cells (43,44).

**Figure 4:**
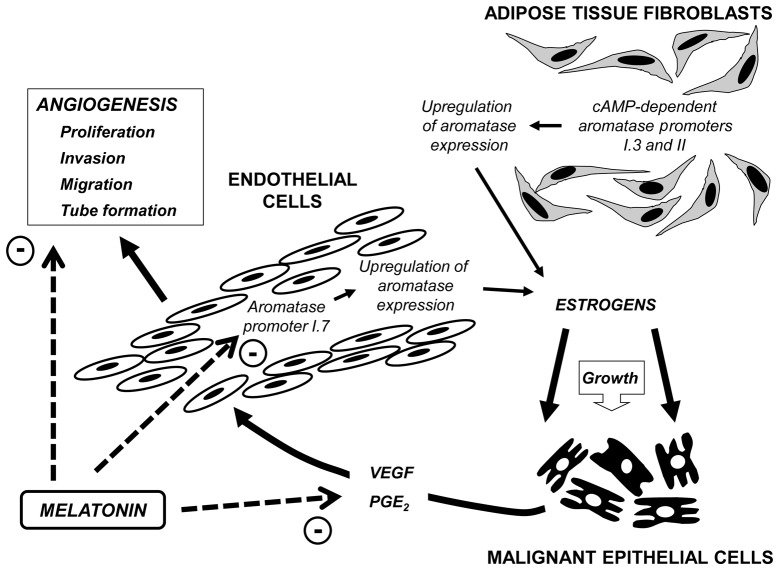
Role of melatonin in the paracrine interactions that occur between malignant epithelial cells and proximal endothelial cells. Melatonin may be important in reducing endothelial cell proliferation, invasion, migration and tube formation, through a downregulatory action on VEGF and PGE_2_. PGE_2_ induced by VEGF may directly promote angiogenesis and melatonin through its downregulatory action on the expression of cyclooxygenase enzymes, decreasing the levels of PGE_2_ and reducing the levels of angiogenesis. Melatonin also inhibits aromatase activity and expression in endothelial cells by regulating the gene expression of specific aromatase promoter region I.7, thereby reducing the local production of estrogens. VEGF, vascular endothelial growth factor; PGE_2_, prostaglandin E_2_.

In endothelial cells, melatonin decreases the aromatase activity and expression mainly by inducing a significant downregulation in aromatase expression specifically driven by promoter I.7, the major promoter directing aromatase expression in endothelial cells (Fig. 4) (45).

VEGF, a major regulator of endothelial growth, added to endothelial cell cultures stimulates the proliferation of these cells and melatonin counteracts this effect (46). Melatonin reduces VEGF mRNA expression in human breast cancer (MCF-7) cells and also reduces VEGF levels in cell culture media of malignant epithelial cells (Fig. 4). Cocultures of breast malignant epithelial cells and endothelial cells is an approach to simulate *in vitro* the paracrine interaction between these cells in the mammary tumors. The presence of malignant epithelial cells in the cocultures is able to stimulate the endothelial cell proliferation and increase the VEGF levels in the culture media. Melatonin counteracts the stimulatory effects on endothelial cell proliferation and on VEGF protein levels in the coculture media. The changes in endothelial cell proliferation induced by melatonin are mediated by an inhibition of the synthesis of VEGF in malignant epithelial cells. Conditioned media from malignant cells stimulate endothelial cell proliferation, and this effect is significantly counteracted by anti-VEGF and melatonin (46).

All these findings suggest that melatonin may play a role in the paracrine interactions between malignant epithelial cells and proximal endothelial cells, through a downregulatory action on VEGF expression in human breast cancer cells, which decreases the levels of VEGF surrounding endothelial cells. Lower levels of VEGF may be important in reducing the number of estrogen-producing cells proximal to malignant cells, as well as in decreasing tumoral angiogenesis. Antiangiogenic activity of melatonin against the pro-angiogenic effects of breast cancer cells has also been described (47). Recently, it has been demonstrated that melatonin has effects on different steps of the angiogenic process in endothelial cell cultures (47). Melatonin strongly inhibits the proliferation of endothelial cells and counteracts the stimulatory effect induced by estradiol. In Transwell assays, melatonin has been identified to reduce the number of endothelial cells that invaded through a basement membrane in response to VEGF. Endothelial cell migration is essential for the formation of new blood vessels during neo-angiogenesis. Melatonin treatment strongly inhibits the migration of endothelial cells in wound-healing assays. Another important step during neo-angiogenesis is the formation of tubes by endothelial cells. It is established that VEGF increases the formation of a branching network of tubes. Melatonin disrupts the tube formation and counteracts the VEGF-stimulated tubular network formation by endothelial cells. In addition, conditioned media collected from breast cancer cells are angiogenically active and stimulate tubule length formation. This effect is significantly counteracted by the addition of either anti-VEGF antibody or melatonin, which suggests that the melatonin-induced decrease of capillary structure formation stimulated by conditioned media from MCF-7 cells may occur as a result of inhibition of VEGF activity (47).

Melatonin may play a role in the paracrine interactions that take place between malignant epithelial cells and proximal endothelial cells, acting by different mechanisms. On one hand, melatonin exerts antiangiogenic effects and may be important in reducing endothelial cell proliferation, invasion, migration and tube formation, through a downregulatory action on VEGF and PGE_2_ (Fig. 4). PGE_2_ synthesis induced by VEGF may directly promote angiogenesis and melatonin through its downregulatory action on the expression of COX enzymes, which decrease the levels of PGE_2_ and reduce angiogenesis. On the other hand, melatonin inhibits aromatase activity and expression in endothelial cells by regulating gene expression of specific aromatase promoter regions, thereby reducing the local production of estrogens (Fig. 4).

## 5. Conclusions

Several lines of evidence highlight the contribution of the tumor microenvironment to its growth and maintenance. Cells immediately adjacent to the tumor are not only passive structural support but also active elements in tumor progression. Among the numerous different cell types surrounding breast cancer cells, the most abundant are those that compose mammary adipose tissue. Ninety percent of these resident cells of adipose tissue are fibroblasts, the precursors of mature adipocytes, and 7% are endothelial cells (35). Epithelial-stromal interactions in breast tumors inhibit adipogenic differentiation and enhance estrogen formation by increasing the aromatase activity of the undifferentiated fibroblasts. All these actions are mediated by cytokines, such as TNF-α, IL-11 and IL-6, produced by malignant epithelial cells. Melatonin may reduce the formation of undifferentiated fibroblasts surrounding malignant epithelial cells by stimulating the differentiation of fibroblasts to mature adipocytes and adipogenesis, and by decreasing the aromatase activity of the fibroblasts and adipocytes through a downregulatory action on the expression of antiadipogenic cytokines, which decrease the levels of these cytokines. Lower levels of TNF-α, IL-6 and IL-11 stimulate the differentiation of fibroblasts and decrease the aromatase activity and expression. Melatonin also decreases the production of PGE_2_ by malignant cells, which upregulates aromatase expression both in the tumor itself and in the surrounding adipose tissue and enhances the production of IL-11 by tumor cells. Endothelial cells also produce estrogens from androgens precursors. Melatonin decreases the activation of promoter I.7 and results in decreased aromatase expression. In addition, melatonin reduces endothelial cell proliferation, invasion, migration and tube formation, through a downregulatory action on VEGF. This melatonin modulation of epithelial-stromal interactions favors lower numbers of undifferentiated fibroblasts, angiogenesis and reduced local estrogen concentrations in breast tumors.

Melatonin may play a role in the paracrine interactions that occur between malignant epithelial cells and proximal adipose and endothelial cells, through a downregulatory action on cytokines and growth factors produced by breast tumor cells. The actions of melatonin described in the present review involve antiproliferative, antiaromatase and antiangiogenic effects, and suggest that melatonin may potentially be beneficial as an anticancer drug in the prevention and treatment of estrogen-dependent mammary tumors. Therefore, this creates interesting possibilities for the clinical applications of melatonin in breast cancer.
